# A deep learning approach for EPID-based patient-specific quality assurance incorporating daily machine-state variations

**DOI:** 10.3389/fonc.2026.1911466

**Published:** 2026-10-20

**Authors:** Kihong Pak, Sung-woo Kim, Kyeongyun Park, Byungchul Cho, Yeon Joo Kim, Si Yeol Song, Chiyoung Jeong, Jungwon Kwak

**Affiliations:** 1 Department of Radiation Oncology, Asan Medical Center, Seoul, Republic of Korea; 2 Department of Radiation Oncology, Asan Medical Center, University of Ulsan College of Medicine, Seoul, Republic of Korea

**Keywords:** deep learning, electronic portal imaging device, gamma passing rate, generative adversarial network, machine performance check, patient-specific quality assurance

## Abstract

Patient-specific quality assurance (PSQA) based on electronic portal imaging devices (EPIDs) is widely used for pretreatment verification, but it is usually performed as a single check of the approved plan and reflects the daily state of the linac only to a limited extent. We propose a deep learning approach that predicts the measured EPID image from the calculated portal dose image using the daily machine state as a conditioning input. A Pix2Pix conditional generative adversarial network was trained to translate Anisotropic Analytical Algorithm portal dose images into measured EPID images, with a 235-dimensional Machine Performance Check (MPC) feature vector added as a conditioning input. Synthetic multi-leaf collimator deviation samples were also introduced during training to represent positioning offsets. Six model variants were evaluated across multiple anatomical sites, combining two prediction targets (the EPID image or the dose-difference map) with three input configurations (baseline, MPC, and MPC with deviation augmentation). Gamma analysis was performed at 3%/3 mm, 3%/2 mm, and 2%/2 mm. The difference-target model with full MPC integration performed best, reaching a root-mean-square error of 0.48 percentage points (pp) and a Pearson correlation of 0.944 for the gamma passing rate (GPR) at 3%/2 mm. On perturbed plans, adding MPC features together with deviation-aware augmentation reduced the absolute GPR prediction error by 42%. Incorporating measured machine-state information improved EPID-based PSQA prediction under the MLC perturbations evaluated in this study. These findings suggest the potential value of a fraction-level, machine-state-aware PSQA, in which the pretreatment measurement establishes a baseline and subsequent daily MPC data are used to update the expected GPR of the approved plan on the day each fraction is delivered. Further prospective evaluation of clinically relevant error detection and false-negative risk is warranted. Nevertheless, this approach may provide a basis for extending PSQA from a single pretreatment assessment toward fraction-level monitoring of patient-specific delivery quality during the treatment course.

## Introduction

Advanced radiation therapy techniques such as intensity-modulated radiation therapy and volumetric modulated arc therapy (VMAT) deliver highly conformal dose distributions (1) through complex modulation of multi-leaf collimator (MLC) sequences and gantry motion. The geometric and dosimetric complexity of these techniques makes the delivered dose sensitive to small deviations in delivery parameters, which may compromise the agreement between planned and delivered dose. Patient-specific quality assurance (PSQA) has therefore become standard practice for pretreatment verification that delivery agrees with the treatment plan within acceptable tolerances.

Conventional measurement-based PSQA workflows employ ion chamber arrays, diode arrays, or electronic portal imaging devices (EPIDs) to compare measured and calculated dose distributions. This comparison is typically performed through gamma analysis, as recommended by the American Association of Physicists in Medicine (AAPM) Task Group 218 (TG-218) (2). EPID-based portal dosimetry has been widely adopted because the imager is integrated into the linac, enabling efficient acquisition of two-dimensional portal dose images without additional setup, as reviewed in AAPM TG-307 (3). While alternative calculation-based PSQA approaches such as independent dose recalculation (4) and trajectory log-file analysis (5) have been proposed, EPID-based methods retain the unique advantage of capturing the actually delivered fluence with spatial resolution, enabling not only pass/fail evaluation but also localization of the failure region.

Despite its widespread adoption, EPID-based PSQA is typically performed as a one-time pretreatment check of the approved plan. While this provides an important independent verification of patient-specific delivery, it offers limited ability to reflect the daily state of the linac when the PSQA result is interpreted. The workflow also carries practical costs, since every plan is verified by physical measurement, which adds to clinical workload and deposits dose on the imager panel that contributes to cumulative radiation damage of the detector. In routine practice, even when the machine operates within standard QA tolerances, small variations in MLC positioning, beam output, field geometry, or imager response can influence the agreement between the planned and the delivered fluence. Accounting for these daily variations could therefore extend PSQA from a single plan-level check toward an assessment that reflects the machine state on the day each fraction is delivered. Recent advances in machine learning have made such prediction feasible, particularly through generative models capable of learning the mapping between calculated and measured dose distributions.

Several studies have investigated machine learning approaches to PSQA prediction. Earlier work, including those by Valdes et al. (6, 7), Lam et al. (8), and Zhu et al. (9), predicted gamma passing rates (GPR) directly from plan complexity features using tree-based or shallow learning models. More recent studies have used deep neural networks trained on full two-dimensional portal dose images to predict either the EPID image or the gamma distribution, including the work of Huang et al. (10), Yoganathan et al. (11), Dal Bosco et al. (12), and Tozuka et al. (13). Most relevant to the present work, Matsuura et al. (14) employed Pix2Pix (15), a conditional generative adversarial network (cGAN) (16, 17), to synthesize the gamma distribution directly per criterion, achieving promising pixel-level error-detection performance. While these approaches have substantially advanced PSQA prediction, they typically focus on either GPR-level or pixel-level evaluation under a single tolerance. In addition, the integration of daily machine-state information has remained limited, despite being routinely available in modern linacs.

In this study, we propose a deep learning approach for EPID-based PSQA that combines image-to-image translation with feature-level conditioning on the daily machine state and deviation-aware training augmentation. The approach predicts the measured EPID image from the calculated portal dose image using a Pix2Pix model, enabling gamma evaluation across multiple criteria at inference time. Machine Performance Check (MPC; Varian Medical Systems, Palo Alto, CA) features, which are obtained from a routine daily linac QA procedure, are incorporated as an additional input channel to account for daily variations in linac performance, and synthetic MLC-deviation augmentation is introduced during training to provide the model with examples of MLC positioning offsets and their dosimetric consequences. Two output target representations, the measured EPID image and the dose-difference map, are compared within the same approach, isolating the contribution of each design factor to multi-site PSQA prediction and deviation-detection performance.

## Materials and methods

### Patient datasets

The dataset comprised 50 patients treated with VMAT on a Halcyon system between April 2023 and October 2024 whose pretreatment PSQA was performed with the EPID. Patients were selected retrospectively without predefined selection criteria, and the site distribution reflects the cases available during this period, with prostate cases forming the largest subgroup. The final cohort comprised head-and-neck (n=8), pelvis (n=34, including the 26 prostate cases), thorax (n=6), and extremities (n=2). Prescribed doses ranged from 20 to 74 Gy in 4 to 37 fractions. All plans consisted of two or three arcs. Excluding two arc fields omitted during data export, 135 arc fields remained (37 patients with three, 11 with two, and 2 with one), delivered with the 6 MV flattening-filter-free beam at a nominal maximum dose rate of 800 MU/min. Because the aperture of each arc is shaped by the MLC and varies with the control point, the field size of each arc was characterized by the irradiated area above 10% of the maximum in the integrated portal dose image, which ranged from 26 to 284 cm² (equivalent square 5.1 to 16.9 cm, median 12.6 cm). All treatment plans were created using the Eclipse v15.6.8 treatment planning system (Varian Medical Systems, Palo Alto, CA), with volumetric dose calculations and 2D portal dose images per arc field generated by Anisotropic Analytical Algorithm (AAA) v15.6.06 on a 2.5 mm calculation grid, replacing the legacy Portal Dose Image Prediction algorithm. PSQA measurements were performed on a Halcyon system v3.0 using a 6 MV flattening-filter-free beam, with a dual-layer MLC consisting of a distal layer of 28 leaf pairs and a proximal layer of 29 leaf pairs, where distal and proximal refer to the distance from the source. The leaves are 1.0 cm wide at the isocenter and the two layers are staggered by half a leaf width, giving 57 leaves per bank and a maximum field size of 28 × 28 cm². Because both layers contribute to field shaping, the MPC reports the positions of all 57 leaves of each bank, with leaf numbers 1 to 28 denoting the distal layer and 29 to 57 the proximal layer.

The corresponding measured EPID images were acquired with an amorphous silicon (a-Si) panel (active area 43 × 43 cm², pixel pitch 0.336 mm). The EPID was calibrated according to the vendor procedure, comprising dark field and flood field acquisition, update of the pixel defect map, and dosimetric calibration of the panel response. These calibrations were not repeated on a fixed schedule but were performed whenever the mean dose difference of routine EPID PSQA measurements showed a systematic drift. On the Halcyon system, the MV imager is fixed to the ring gantry and no separate imager positioning calibration was performed. The daily constancy of the imager gain and uniformity was monitored by the MPC procedure described in the following subsection. The original AAA portal dose images ranged from 534 × 534 to 616 × 616 pixels at 0.649 mm resolution, whereas the measured EPID images had a fixed matrix of 1280 × 1280 pixels at 0.218 mm resolution. To ensure consistency in model training, all images were resampled to a uniform spatial resolution of 1 mm and a matrix size of 256 × 256 pixels, and normalized to their respective maximum pixel values prior to gamma analysis. Spatial data augmentation (rotation, translation, cropping) was not applied, since the dataset used only a limited number of fixed collimator angles where minor coordinate-level alterations could introduce uncertainty in subsequent GPR estimation.

### Machine Performance Check features

The MPC procedure is an automated daily QA routine performed on the Halcyon system using the Varian Isocal phantom. Each MPC session evaluates a comprehensive set of machine parameters covering isocenter accuracy, beam output and uniformity, collimation (MLC-related features, field edges, and collimator rotation), gantry and couch positioning, and MV imager calibration. The Halcyon MPC system records 253 individual parameters in total, archived in CSV format on each treatment day.

To incorporate the daily machine state into the model, a subset of 235 MPC parameters was selected from the full 253-parameter set based on their expected influence on EPID image formation. The selected subset comprises 228 MLC-related features (Leaf A and Leaf B positions at 57 leaves each, together with their backlash counterparts) and 7 additional machine-state features covering beam output and uniformity, field edges (Y1 and Y2), collimator rotation offset, and EPID calibration gain and uniformity. Parameters that do not change the portal dose pattern projected onto the EPID during PSQA were excluded. On the Halcyon system, the EPID is mounted on the ring gantry opposite the source, so the source-to-imager geometry is fixed and rotates together with the gantry. The gantry angle deviations (Gantry Absolute and Gantry Relative) were therefore excluded, because a gantry angle offset changes the beam direction in room coordinates but not the fluence pattern projected onto the panel. The couch position items, the isocenter size, and the kV isocenter offset were excluded because no patient or phantom is placed at the isocenter and the comparison between the calculated and measured portal dose images is performed in the MV imager coordinate frame. The MV isocenter offset can in principle shift the entire portal dose image on the panel. This item was nevertheless excluded because its daily tolerance of 0.5 mm is below the 1 mm resampling grid used in this study and well within the distance-to-agreement criteria of 2 to 3 mm. In addition, such a rigid shift does not change the shape of the fluence pattern. The kV field edge items describe the kV imaging system rather than the MV beam. The MU1 and MU2 gain items describe the cumulative gain change of the two monitor ion chambers, whose net effect on the dose delivered per MU is already represented by the retained Beam Output Change item. In total, 18 of the 253 items were excluded, and the retained items are listed in **Table 1**.

**Table 1:** Composition of the 235-dimensional MPC feature vector.

Group	Item [unit]	n
Beam Group	Beam Output Change [%]	1
	Beam Uniformity Change [%]	1
Collimator (MLC)	MLC Leaf 1 to 57, Bank A [mm]	57
	MLC Leaf 1 to 57, Bank B [mm]	57
	MLC Backlash Leaf 1 to 57, Bank A [mm]	57
	MLC Backlash Leaf 1 to 57, Bank B [mm]	57
Collimator (Field edges)	Field Edge Y1 [mm]	1
	Field Edge Y2 [mm]	1
Collimator	Collimator Rotation Offset [°]	1
MV Imager	MV Imager Calibration Gain [%]	1
	MV Imager Calibration Uniformity [%]	1
**Total**		**235**

Items of the daily MPC report retained as model inputs are listed with the number of features per item. The 18 excluded items (isocenter, gantry, couch, kV field edge, and MU chamber gain) are described in the text.

Bold marks the total row.

For each arc field, the MPC session recorded on the same day as the corresponding EPID measurement was retrieved from the institutional archiving server. Each selected parameter was normalized by dividing its recorded deviation by the MPC tolerance reported for that parameter. The two EPID calibration items (gain and uniformity), for which the MPC export does not provide a tolerance, were normalized by their nominal full-scale value of 100%. This tolerance-based normalization expresses each parameter as a fraction of its own reference scale, so that features with different units (mm, %, and degrees) and different dynamic ranges contribute on a comparable scale rather than being dominated by parameters with wider numerical ranges. Under this scaling, all normalized values fell within ±1, consistent with every parameter passing its daily tolerance. The 235 normalized values were then concatenated into a one-dimensional vector that served as the machine-state input to the network.

### Network architecture

#### GAN-based image-to-image translation framework

A Pix2Pix model, a variant of the cGAN, was employed to translate AAA portal dose images into the corresponding measured EPID images. A generative adversarial network (GAN) consists of two neural networks — a generator that synthesizes images and a discriminator that distinguishes synthesized from real ones — trained jointly in an adversarial manner. Unlike conventional GANs, which generate images from random noise, Pix2Pix conditions both networks on paired input and target images, making it well-suited for image-to-image translation where input and output share the same spatial structure. In this study, the generator was configured to predict, from an AAA portal dose image, either the corresponding measured EPID image or the dose-difference map (AAA portal dose − measured EPID); both target representations were evaluated within the same approach, as described in the Experimental design subsection. The overall architecture is illustrated in **Figure 1**.

**Figure 1:**
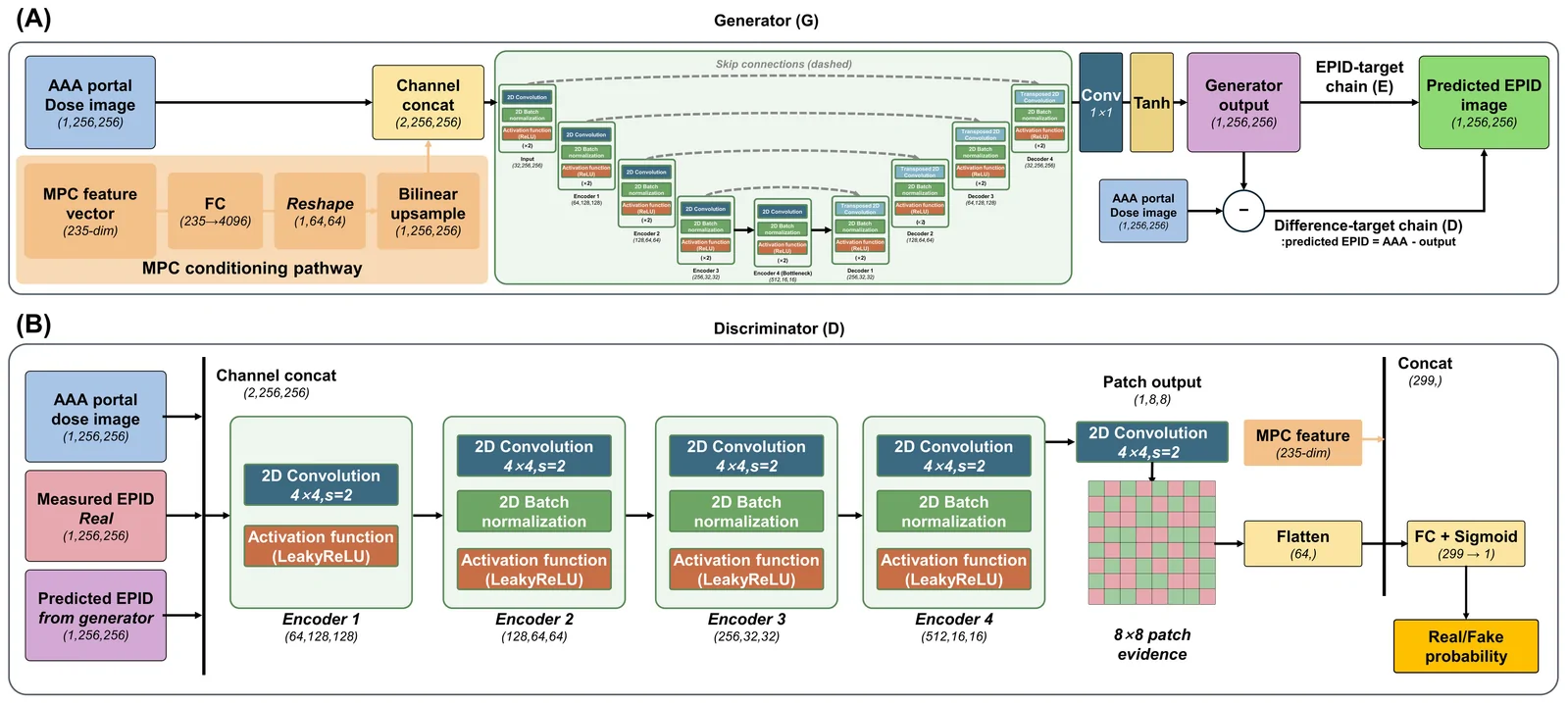
GAN architecture for EPID image prediction. **(A)** Generator: a five-level U-Net that takes the AAA portal dose image and a 235-dimensional MPC feature vector as inputs and outputs a (1, 256, 256) prediction map (EPID image or dose-difference map, depending on the target chain). **(B)** Discriminator: a hybrid PatchGAN whose 8×8 patch evidence map is combined with the MPC feature vector in the final classification head.

#### Generator architecture: U-Net with MPC conditioning

The generator was implemented using a U-Net architecture. The skip connections between the encoder and decoder allow low-level spatial information to bypass the bottleneck and directly inform the output reconstruction, ensuring the preservation of fine-grained dosimetric features such as MLC leaf edges and high-gradient dose regions. The network was constructed with a five-level encoder–decoder depth, with channel counts of 32, 64, 128, 256, and 512 (the deepest level serving as the bottleneck). Each encoder and decoder stage consisted of two consecutive 3×3 convolutions, each followed by batch normalization and ReLU activation. Downsampling used 2×2 max-pooling, and upsampling used 2×2 transposed convolution, with skip connections concatenated channel-wise at each decoder stage. A final 1×1 convolution with Tanh activation produced the (1, 256, 256) generator output.

When MPC features were included, they were provided to the generator as an additional input together with the dosimetric image, so that the predicted output could depend on the measured machine state. This use of the machine state to condition the prediction is referred to as MPC conditioning. The 235-dimensional MPC feature vector was projected through a fully connected layer to a 4096-dimensional embedding. The embedding was reshaped to (1, 64, 64) and bilinearly upsampled to (1, 256, 256). The resulting feature map was channel-stacked with the AAA portal dose image to form a two-channel input to the U-Net generator. This design preserves spatial alignment between the dosimetric image and the machine-state information, while enabling the network to learn how variations in MPC features modulate the predicted output. For configurations without MPC conditioning, only the single-channel AAA portal dose image was used as input. In the EPID-target chain, the generator was trained to directly approximate the measured EPID image. In the difference-target chain, the generator was instead trained to approximate the dose-difference map, and the predicted EPID was reconstructed at inference by subtracting the generator output from the AAA portal dose image. The generator architecture and the MPC conditioning pathway are illustrated in **Figure 1A**.

#### Discriminator and training scheme

The discriminator combined a PatchGAN convolutional backbone with a fully connected classification head that incorporated the MPC feature vector. Following the PatchGAN design, the AAA portal dose image was channel-concatenated with either the measured EPID image (real pair) or the generator-predicted EPID image (fake pair) and processed through five 4×4 stride-2 convolutions (with LeakyReLU activations and BatchNorm, except for the first and final convolutions), producing an 8×8 patch evidence map. By focusing on local patch-level fidelity, this convolutional backbone encourages the generator to reproduce high-frequency dosimetric features such as the steep gradients at MLC leaf edges. The patch evidence map was then flattened to a 64-element vector and concatenated with the 235-dimensional MPC feature vector, and the resulting 299-dimensional vector was passed through a fully connected layer with sigmoid activation to yield a single real/fake probability per sample. This MPC fusion in the classification head allowed the discriminator’s decision boundary to be informed by the machine state in addition to image-level fidelity. The discriminator architecture is illustrated in **Figure 1B**.

All model variants were trained using the standard Pix2Pix learning scheme. The generator loss combined an adversarial term with a weighted L1 reconstruction term (L1 weight = 100), and the discriminator was trained with a binary cross-entropy adversarial objective. Both networks were optimized using the Adam optimizer with a learning rate of 10^-5^ and a batch size of 4, implemented in PyTorch 2.4.0 (18). The number of epochs was fixed in advance at 2000, informed by the 3000 epochs used by Matsuura et al. (14), and the final checkpoint was used for all evaluations without validation-based model selection. The random seed was not fixed in these training runs. To quantify the resulting run-to-run variability, the same configurations were retrained with five fixed random seeds under identical hyperparameters (RTX 4090, approximately 33 and 26 minutes per 2000-epoch run for the MPC-conditioned and base configurations, respectively). In these retraining runs, the generator L1 loss decreased steeply during the first approximately 500 epochs, whereas the decrease between epochs 1000 and 2000 was about 5 × 10^-4^ relative to the [0,1] normalized-intensity scale, indicating that the fixed budget of 2000 epochs was conservative. These hyperparameters were held identical across all six model variants to ensure that performance differences reflect input/output configuration rather than training-side variability.

### Experimental design

To quantify the contribution of each design factor to PSQA prediction performance, six model variants were constructed and evaluated along two orthogonal design axes. The first axis specifies the prediction target, with the generator predicting either the measured EPID image directly (the EPID-target chain) or the dose-difference map (the difference-target chain). The second axis specifies the input and training configuration through three progressive levels. The baseline (Base) uses only the AAA portal dose image as input and is trained on the normal dataset. The MPC level adds the MPC feature vector as a second input channel. The MPC+aug level further adds synthetic MLC-deviation samples to the training set. The combination of these two axes yields six model variants, designated as Base-E, MPC-E, and MPC+aug-E for the EPID-target chain and Base-D, MPC-D, and MPC+aug-D for the difference-target chain. The model definitions are summarized in **Table 2**.

**Table 2:** Definition of the six model variants used in this study.

Model	Output target	MPC features	MPCmod augmentation	n train	n test
Base-E	EPID image	–	–	107	28
MPC-E	EPID image	✓	–	107	28
MPC+aug-E	EPID image	✓	✓	133	38*
Base-D	Difference map	–	–	107	28
MPC-D	Difference map	✓	–	107	28
MPC+aug-D	Difference map	✓	✓	133	38*

The fair-comparison test set (n=28 normal samples) is used for direct cross-model comparison. For MPC+aug models (marked with *), the test set additionally includes 10 MPCmod samples for perturbation sensitivity evaluation.

The 135 normal arc fields were acquired from 50 patients and split into 107 training and 28 test samples. Arcs from a given patient were kept within a single subset, so that every patient was assigned entirely to either the training or the test set. No arc field appeared in more than one subset. The assignment was performed manually at the patient level rather than by random sampling. The training set comprised 39 patients and the test set 11 patients, containing 6 and 2 head-and-neck, 26 and 8 pelvis, 5 and 1 thorax, and 2 and 0 extremity patients, respectively. The 28-sample test set is referred to throughout this paper as the fair-comparison test set. The Base and MPC configurations (Base-E, MPC-E, Base-D, MPC-D) were trained on the 107 normal training samples. The MPC+aug configurations (MPC+aug-E, MPC+aug-D) additionally incorporated 26 synthetic MLC-deviation samples in their training data, with weighted random sampling applied to balance their exposure during training. The rationale for this sampling strategy and the construction of the synthetic deviation samples are detailed in the following subsection.

Because the MPC+aug models were trained on a superset of the data used by the Base and MPC models, their respective test sets would not be directly comparable. To enable a fair comparison across the six model variants, all primary cross-model evaluations were performed on the 28-sample fair-comparison test set, which was held out from training in every configuration. For the four configurations that incorporate MPC features (MPC-E, MPC+aug-E, MPC-D, MPC+aug-D), each test sample was paired with its corresponding 235-dimensional MPC vector at inference time. Perturbation sensitivity, in contrast, was evaluated separately on a plan-paired test set of 16 samples, comprising 10 perturbed samples and the 6 original arcs from which they were derived.

### MPCmod synthetic-deviation dataset

#### Synthetic MLC-deviation augmentation

In routine clinical practice, MLC delivery faults are infrequent because daily mechanical QA, including the MPC procedure (19), detects deviations before treatment, triggering intervention when tolerances are exceeded. As a consequence, a training set drawn from clinically delivered plans contains few examples with large discrepancies between the measured EPID and the AAA prediction. Such an imbalance limits the model’s ability to learn how machine-state perturbations propagate to the EPID and to the GPR. To address this limitation, a controlled synthetic-deviation dataset was constructed. Deliberate MLC positioning offsets were introduced into normal plans, and the perturbed plans were re-delivered and re-measured on the EPID. The corresponding MPC feature vectors were then modified to reflect the same offsets. We refer to this combined plan-and-MPC modification protocol as MPCmod augmentation. This procedure produces paired (input, MPC, EPID) sets in which the MPC vector and the measured EPID drift consistently with one another, thereby providing the model with examples of how machine-state changes translate into dosimetric deviations.

To generate the synthetic-deviation dataset, six base arc fields (two per plan, from one head-and-neck, one thorax, and one pelvis plan) were selected from the institutional cohort. Six preset MLC offset patterns of ±1 mm were applied to these base plans through direct modification of the leaf positioning sequences in the DICOM-RP files (**Table 3**), spanning asymmetric single-bank shifts, symmetric aperture-narrowing or -widening translations, and localized offsets restricted to selected leaves. In Patterns 5 and 6, the offset was applied to leaves 12, 13, and 17 of the distal layer and to leaves 40, 41, and 45 of the proximal layer, which overlap the same lateral positions offset by half a leaf width because of the stagger, so that the localized offset affected both layers at overlapping positions. These positions lie within about 3.5 cm of the central axis, a region covered by most clinical arcs, and the combination of an adjacent pair (leaves 12 and 13) and an isolated leaf (leaf 17) produces two forms of localized perturbation, a contiguous two-leaf offset and a single-leaf offset. The offset magnitude of 1 mm was chosen to match the widely used tolerance for MLC leaf positional accuracy recommended in AAPM TG-142 (20), so that the synthetic faults represent deviations at the boundary of clinical action levels. Applying each pattern to each base arc field yielded 36 perturbed arc fields, which were physically re-delivered and re-measured on the Halcyon system using the same protocol as the normal cohort. These were partitioned into 26 training and 10 test samples.

**Table 3:** Preset MLC leaf offset patterns used to generate synthetic positioning offsets.

Pattern	Bank A leaves	Bank A offset (mm)	Bank B leaves	Bank B offset (mm)
1	All	0	All	−1
2	All	+1	All	0
3	All	−1	All	+1
4	All	+1	All	−1
5	12, 13, 17, 40, 41, 45	−1	12, 13, 17, 40, 41, 45	+1
6	12, 13, 17, 40, 41, 45	+1	12, 13, 17, 40, 41, 45	−1

Bank A and Bank B refer to the right and left leaf banks, respectively, from the perspective of a patient in the supine position on the Halcyon system. Patterns 1 and 2 represent asymmetric single-bank shifts; Patterns 3 and 4 represent symmetric translations producing aperture-widening and aperture-narrowing biases, respectively; Patterns 5 and 6 represent localized offsets restricted to selected leaves.

For each synthetic-deviation sample, the corresponding 235-dimensional MPC vector was constructed by taking the MPC measurement recorded on the same day as the EPID re-measurement and adding the leaf-by-leaf modification pattern to the appropriate MLC entries. The 36 perturbed arc fields were re-delivered on two treatment days, following the clinical schedule of the treatment machine. All six patterns and both arcs of a given plan were measured within the same day, with the pelvis and thorax plans on the first day and the head-and-neck plan in the second, so the synthetic-deviation vectors reference two different MPC sessions. The 235-dimensional MPC vector comprises 228 MLC-related features (Leaf A and Leaf B positions at 57 leaves each, plus their backlash counterparts) and 7 additional machine-state features (beam output and uniformity, field edges Y1 and Y2, collimator rotation offset, EPID calibration gain and uniformity). Specifically, the same ±1 mm offset that was applied to the leaf positioning sequence was added, in millimeters prior to the tolerance normalization, to the corresponding MPC leaf-position entries of Bank A and Bank B and to their backlash counterparts, consistent with the grouping used in the feature-masking analysis. The remaining 7 machine-state features were retained at their measured values. This construction ensures that the MPC vector and the measured EPID image are perturbed in a physically consistent manner, allowing the model to receive paired machine-state and dosimetric-outcome data from which it can learn the influence of MLC perturbations on the predicted dose distribution.

During training, weighted random sampling was applied to balance deviation and normal exposure within the 133-sample MPCmod training set. Each deviation sample was assigned a sampling weight of w=2 relative to a weight of 1 for normal samples, raising the per-epoch exposure to deviation samples from approximately 19.5% (uniform sampling) to 32.7%.

#### Plan-paired test set

To evaluate perturbation sensitivity while controlling for plan-specific variability, each of the 10 perturbed samples was referenced against the unperturbed version of the same base arc. The resulting test set contained 16 samples: the 10 perturbed samples and the 6 unperturbed base arc fields from which they were generated. The paired design enables two clinically relevant evaluations. First, it tests whether the model tracks the GPR drop induced by the synthetic perturbation relative to the unperturbed base. Second, it tests whether incorporating MPC features and MPCmod augmentation degrades performance on the unperturbed base plans.

### Evaluation metrics

#### GPR-level evaluation

Gamma analysis was performed following the standard Low formulation (21), with the AAA portal dose image as the reference and the EPID image (either measured or model-predicted) as the evaluation target. A circular distance-to-agreement search was performed on the native 1-mm pixel grid, and the implementation was vectorized over reference pixels for computational efficiency. The search used 1-mm steps without sub-pixel interpolation. This stringent setting was chosen deliberately, since finely interpolated search concentrates the GPR of clinical plans near 100% even at 2%/2 mm and leaves little variance for training and comparing prediction models, at the cost of absolute values that are conservative relative to clinical portal dosimetry software. Predicted and measured GPRs were computed with identical settings, so all comparisons are internally consistent. The implementation was cross-validated against the open-source package pymedphys v0.40.0 (22), agreeing to within 0.01 percentage points on average under identical 0.1-mm search steps. Three acceptance criteria were applied: 3%/3 mm (institutional clinical standard), 3%/2 mm (tighter criterion in line with TG-218 recommendations), and 2%/2 mm (stringent criterion). All gamma calculations were performed in relative-dose mode with global normalization, and a 10% low-dose threshold was applied to exclude regions outside the treatment field. For each test sample, the predicted and measured GPRs were computed using the model-predicted and physically measured EPID images, respectively, against the same AAA reference. For the difference-target chain, the model-predicted EPID was the reconstruction described above (the AAA portal dose image minus the generator output), so that both prediction targets were evaluated identically in the EPID domain against the same AAA reference. Prediction accuracy across the test cohort was summarized by the root-mean-square error (RMSE), expressed in percentage points (pp), and the Pearson correlation coefficient (r) between predicted and measured GPR.

Statistical analyses were carried out between the Base-D and MPC+aug-D models, considering the primary comparison of interest of this study. On both the fair-comparison and plan-paired test sets, per-sample absolute prediction errors were compared using the exact paired Wilcoxon signed-rank test. For the fair-comparison test set, 95% confidence intervals for RMSE, Pearson’s r, and their between-model differences were estimated by paired bootstrap resampling (10,000 resamples). All tests were two-sided with a significance level of 0.05, and analyses were performed in SciPy (23).

#### Pixel-level evaluation

To quantify perturbation sensitivity at the pixel scale, pixel-level sensitivity, specificity, and accuracy were evaluated following the definitions of Matsuura et al. (14). At each pixel within the 10% threshold mask, a true positive (TP) was defined as gamma failure (γ > 1) in both the predicted and measured maps, a true negative (TN) as gamma pass (γ ≤ 1) in both, a false negative (FN) as a missed failure, and a false positive (FP) as a falsely flagged failure. Pixel counts were accumulated across all evaluation cases prior to computing the metrics, so that pixels from cases with larger failure regions contribute proportionally more to the aggregate. Sensitivity, specificity, and accuracy were then computed as $\frac{TP}{TP+FN}$, $\frac{TN}{TN+FP}$, and $\frac{TP+TN}{TP+TN+FP+FN}$, respectively.

#### Per-scenario evaluation

On the plan-paired test set, two scenario-level metrics were reported. The signed error (predicted−measured GPR) indicates the direction of the model’s deviation, and the absolute error summarizes overall agreement independent of direction. Both metrics were reported separately for the unperturbed base plans and for each MLC modification pattern category (asymmetric single-bank shifts, symmetric translations, and localized offsets).

#### MPC feature-group masking

To interpret the contribution of the MPC features, a group-wise masking analysis was performed on the trained MPC+aug-D model at inference time, without retraining. The 235-dimensional MPC vector was partitioned into five physically meaningful groups based on **Table 1** (beam output and uniformity, MLC-related features, field edges, collimator rotation, and EPID calibration), where the MLC leaf position and backlash entries were combined into a single MLC block because the synthetic deviations modify both consistently. In each condition, the entries of one group or of all non-MLC groups combined were set to zero, which corresponds to the nominal machine state under the tolerance-based normalization, while the remaining entries were left unchanged. The predicted GPR at 3%/2 mm was then recomputed on both test sets and compared against the unmasked baseline, which exactly reproduced the reported predictions. As a control against out-of-distribution effects, each condition was repeated with masked entries replaced by their training-set means, and the two schemes led to consistent conclusions.

## Results

### GPR prediction performance under fair comparison

To compare model performance under identical evaluation conditions, all six model variants were assessed on the same 28-sample fair-comparison test set. RMSE and Pearson’s r between predicted and measured GPR were computed under three gamma criteria. Among the six models, MPC+aug-D achieved the highest Pearson’s r under all three criteria and the lowest RMSE at the 3%/2 mm and 2%/2 mm criteria; at 3%/3 mm, where errors were small for all models, Base-E showed the lowest RMSE, as summarized in **Table 4**. At the 3%/2 mm criterion, MPC+aug-D yielded an RMSE of 0.48 pp and r of 0.944, compared with RMSE 0.69 pp and r 0.872 for Base-D and RMSE 0.55 pp and r 0.876 for Base-E. However, on the fair-comparison test set, this difference between Base-D and MPC+aug-D was within the range of sampling variability, as the between-model differences in RMSE (95% CI −0.05 to 0.52 pp) and r (95% CI −0.06 to 0.10) both included zero, and the per-sample absolute errors did not differ significantly (exact paired Wilcoxon, p = 0.99). Under the more stringent 2%/2 mm criterion, MPC+aug-D maintained an RMSE of 1.27 pp and r of 0.936, while the corresponding values for Base-D and Base-E rose to 2.01 and 1.68 pp with r values dropping to 0.791 and 0.851, respectively. The agreement between predicted and measured GPR at the 3%/2 mm criterion is illustrated for all six models in **Figure 2**.

**Table 4:** GPR prediction performance of the six model variants on the 28-sample fair-comparison test set. RMSE, root-mean-square error of GPR (pp); r, Pearson correlation between predicted and measured GPR.

Model	3%/3 mm		3%/2 mm		2%/2 mm	
	RMSE (pp)	r	RMSE (pp)	r	RMSE (pp)	r
Base-E	**0.11**	0.915	0.55	0.876	1.68	0.851
MPC-E	0.29	0.535	0.77	0.601	1.82	0.691
MPC+aug-E	0.23	0.821	0.81	0.787	1.99	0.708
Base-D	0.16	0.914	0.69	0.872	2.01	0.791
MPC-D	0.18	0.840	0.72	0.732	1.88	0.725
MPC+aug-D	0.13	**0.922**	**0.48**	**0.944**	**1.27**	**0.936**

Bold values indicate the best result in each column.

**Figure 2:**
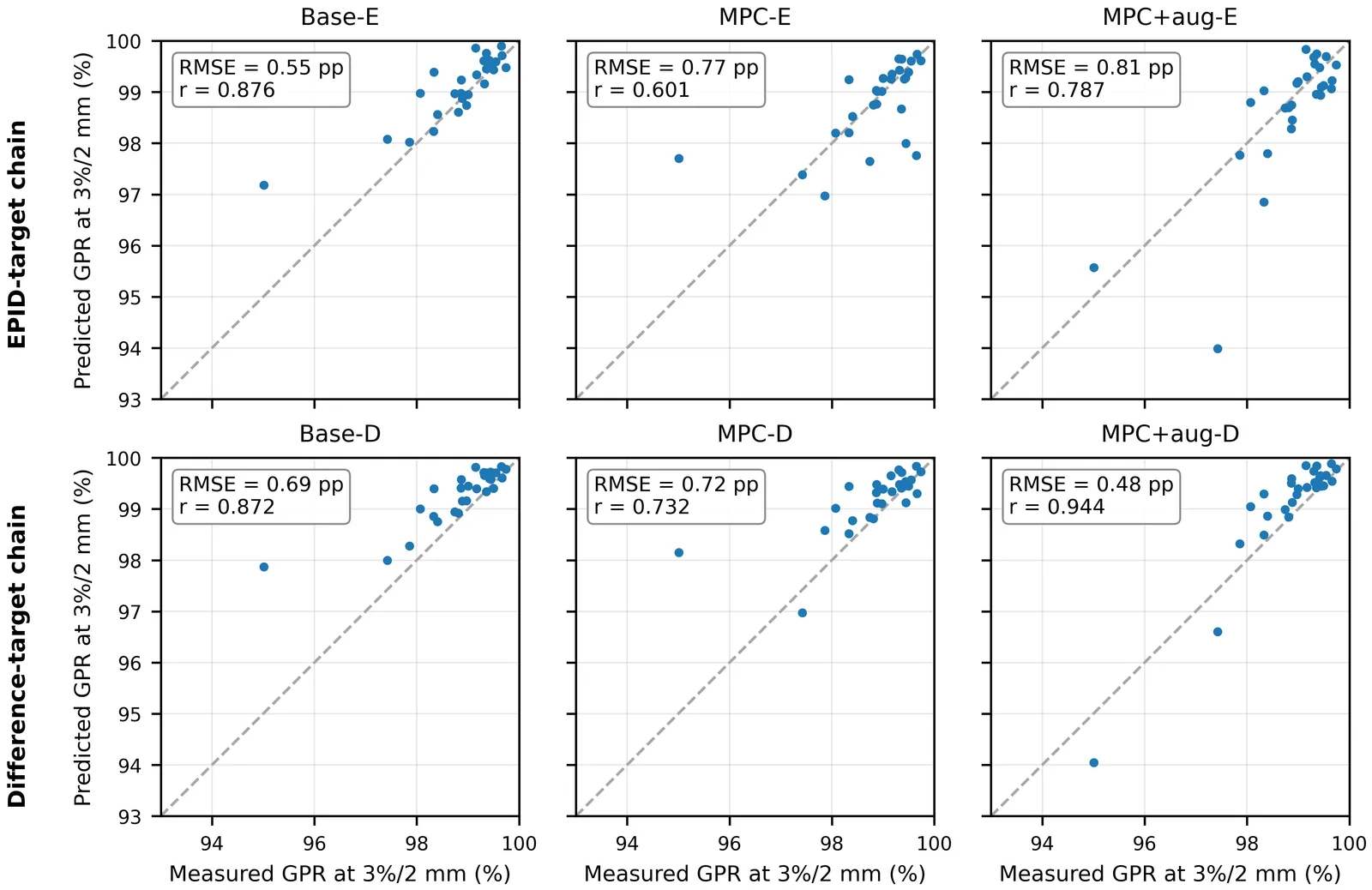
Predicted versus measured GPR at the 3%/2 mm criterion on the 28-sample fair-comparison test set, for the three variants of the EPID-target chain (top) and those of the difference-target chain (bottom). The dashed line denotes perfect agreement. Each panel reports the RMSE in percentage points (pp) and the Pearson correlation coefficient (r).

Within both chains, the addition of MPC features alone produced a small decrease in Pearson’s r relative to the Base configuration, whereas the further addition of MPCmod augmentation recovered the performance. The full configurations yielded r values at 3%/2 mm of 0.787 (MPC+aug-E) and 0.944 (MPC+aug-D), compared with 0.876 (Base-E) and 0.872 (Base-D). In the difference-target chain, the full configuration yielded the numerically best RMSE and r among the six variants, although this improvement over Base-D was not statistically significant on the fair-comparison test set. Across the five retraining seeds, MPC+aug-D yielded an RMSE of 0.60 ± 0.12 pp and an r of 0.937 ± 0.010 at 3%/2 mm (mean ± standard deviation), whereas Base-D showed substantially larger seed sensitivity (r of 0.71 ± 0.24). The reported results correspond to the single originally trained checkpoint, whose RMSE lies at the favorable end of this range.

### Pixel-level performance

To assess perturbation sensitivity at the pixel scale, sensitivity, specificity, and accuracy were computed on the same test set, with pixel counts aggregated across all cases. The results under three gamma criteria are summarized in **Table 5**. MPC+aug-D achieved the highest values across all three metrics under all three criteria, with sensitivity increasing from 27.80% (3%/3 mm) to 29.61% (3%/2 mm) and 32.18% (2%/2 mm). Specificity (97.55–99.88%) and accuracy (95.37– 99.67%) were uniformly high across all six models, with only minor variations that did not reverse the overall ranking. Sensitivity exhibited the largest spread across model variants: within the difference-target chain, sensitivity at 2%/2 mm increased monotonically from 26.05% (Base-D) to 30.05% (MPC-D) to 32.18% (MPC+aug-D), showing a 24% relative gain over Base-D. However, within the EPID-target chain, it increased from 22.75% (Base-E) to 31.28% (MPC-E), then decreased to 25.67% (MPC+aug-E).

**Table 5:** Pixel-level sensitivity, specificity, and accuracy (in percent) of the six model variants on the 28-sample fair-comparison test set.

Model	3%/3 mm Sens/Spec/Acc	3%/2 mm Sens/Spec/Acc	2%/2 mm Sens/Spec/Acc
Base-E	19.74/99.78/99.54	21.02/99.49/98.77	22.75/98.37/96.07
MPC-E	21.71/99.69/99.46	25.16/99.18/98.50	31.28/97.62/95.61
MPC+aug-E	23.48/99.66/99.43	23.54/99.22/98.54	25.67/97.55/95.37
Base-D	19.65/99.86/99.63	22.42/99.64/98.93	26.05/98.72/96.51
MPC-D	23.58/99.85/99.63	25.51/99.57/98.89	30.05/98.34/96.27
**MPC+aug-D**	**27.80/99.88/99.67**	**29.61/99.66/99.02**	**32.18/98.79/96.76**

Bold values indicate the best result in each column.

### Three-way ablation of MPC features and deviation augmentation

To isolate the contributions of MPC conditioning and MPCmod deviation augmentation, the six model variants were further evaluated on the 16-sample plan-paired test set, which comprised 10 perturbed samples and their 6 base arc fields. The mean absolute GPR prediction error on perturbed plans, defined as the mean of the absolute differences between predicted and measured GPR across the 10 perturbed samples under the 3%/2 mm criterion, is reported in **Table 6**. Within the difference-target chain, the absolute error decreased from 3.67 pp (Base-D) to 2.94 pp (MPC-D), and further to 2.12 pp (MPC+aug-D), corresponding to a 42% reduction across the full chain. On the perturbed samples, this reduction in per-sample absolute error from Base-D to MPC+aug-D was statistically significant (exact paired Wilcoxon, p = 0.020). The two stepwise contributions were of comparable magnitude. Adding MPC features alone reduced the error by approximately 20% relative to Base-D, and the further addition of MPCmod augmentation reduced it by an additional 28% relative to MPC-D. The same stepwise ablation along the EPID-target chain produced a smaller improvement, with the absolute error decreasing from 3.45 pp (Base-E) to 3.04 pp (MPC-E) and then to 2.71 pp (MPC+aug-E), corresponding to a 21% reduction across the full chain.

**Table 6:** Mean absolute GPR prediction error (pp) on the 10 perturbed samples of the plan-paired test set at the 3%/2 mm criterion.

Configuration	EPID-target	Difference-target
Base	3.45	3.67
MPC	3.04	2.94
MPC+aug	2.71	2.12

Inspection of the signed errors revealed a further difference between the two chains. Within the EPID-target chain, adding MPC features alone reversed the polarity of the prediction error: Base-E over-predicted the perturbed-plan GPR by +3.45 pp on average, whereas MPC-E under-predicted it by −3.03 pp. This indicates that MPC features in the EPID-target configuration push the predicted EPID intensity downward more aggressively than the measurement supports. The further addition of MPCmod augmentation in MPC+aug-E returned the signed error to +1.94 pp, restoring the original polarity. In contrast, the difference-target chain preserved the sign of the prediction error throughout: Base-D, MPC-D, and MPC+aug-D all over-predicted the perturbed-plan GPR, with the signed error decreasing monotonically from +3.67 to +2.70 to +1.83 pp without reversing into under-prediction. Representative MPC+aug-D predictions for perturbed samples from three anatomical sites are shown in **Figure 3**. The predicted EPID images and the corresponding gamma maps closely reproduce the measured distributions, with the predicted GPR values agreeing with the measured values at the 3%/2 mm criterion.

**Figure 3:**
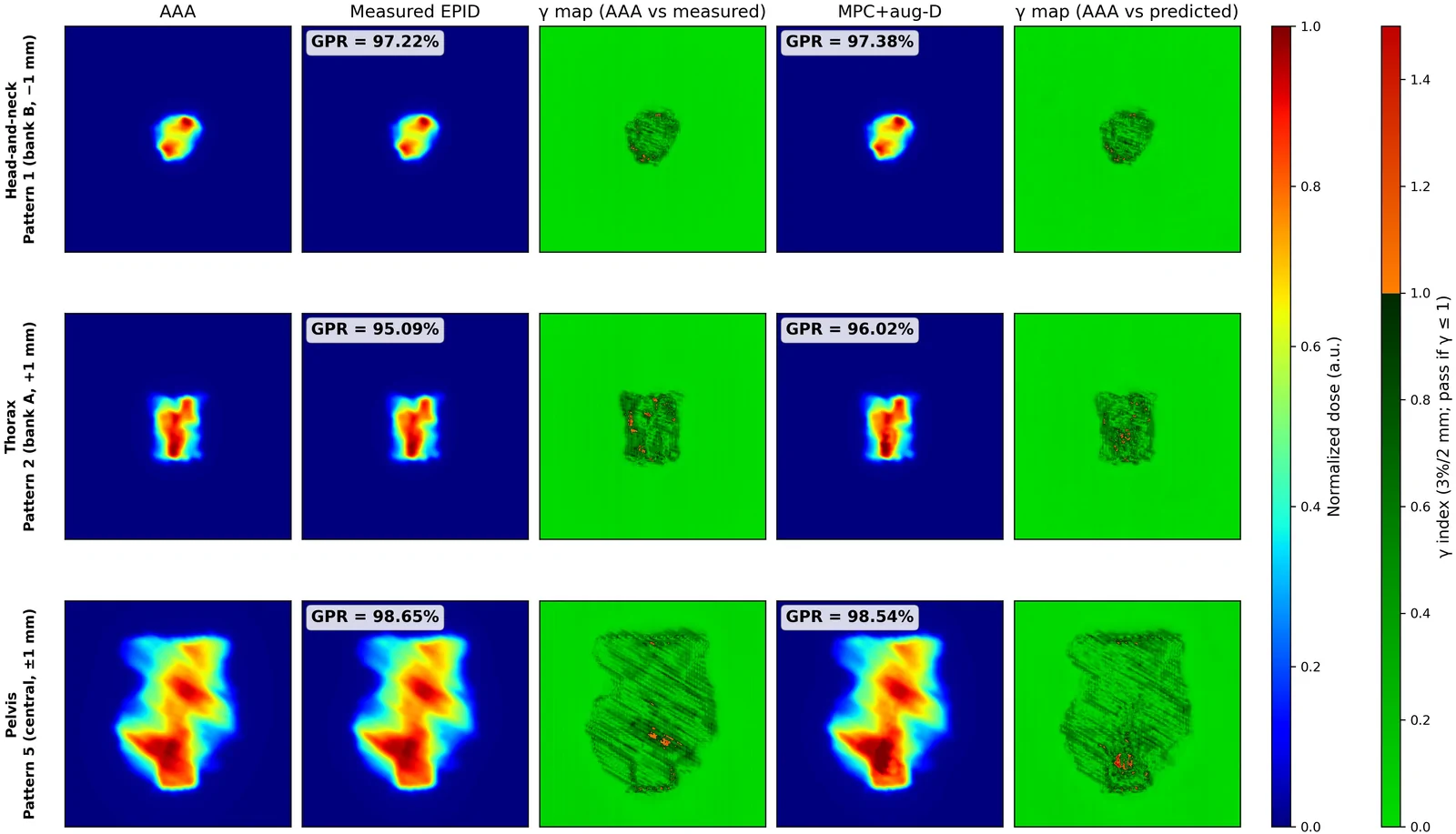
Representative MPC+aug-D predictions for perturbed samples at the 3%/2 mm criterion. Rows correspond to three sites and applied MLC offset patterns (head-and-neck, bank B −1 mm; thorax, bank A +1mm; pelvis, central ±1 mm). The GPR is annotated on each EPID image; colorbars indicate normalized dose (left) and gamma index (right).

### Contribution of MPC feature groups

Group-wise masking localized the fault response of the model to the MLC-related MPC features (**Table 7**). On the 10 perturbed samples, setting the MLC-related features (leaf positions and backlash, 228 of the 235 entries) to their nominal value increased the mean signed error from +1.83 pp to +3.40 pp, approaching the +3.67 pp of Base-D, which uses no MPC input, whereas masking the non-MLC groups, individually or combined, left the fault response essentially unchanged. On the fair-comparison test set, no masking condition changed the RMSE by more than 0.05 pp. The MPC input therefore contributes little to prediction accuracy on unperturbed plans, and its effect is concentrated in the MLC features under perturbed delivery, consistent with the statistical comparison above.

**Table 7:** Group-wise MPC feature masking of the MPC+aug-D model at the 3%/2 mm criterion.

Masking condition	Fair-comparison test set (n = 28)		Plan-paired test set (n = 10)	
	RMSE (pp)	r	Signed error (pp)	MAE (pp)
None (baseline)	0.48	0.944	+1.83	2.12
Beam output/uniformity	0.49	0.942	+1.89	2.17
MLC-related features	0.47	0.957	+3.40	3.40
Field edges	0.45	0.944	+1.83	2.09
Collimator rotation	0.52	0.938	+1.80	2.06
EPID calibration	0.48	0.944	+1.82	2.11
All non-MLC groups	0.45	0.944	+1.93	2.16

In each condition the listed group was set to its nominal value (zero deviation) at inference, with the remaining entries unchanged. RMSE and r are evaluated on the 28-sample fair-comparison test set, and the signed error and mean absolute error (MAE) are averaged over the 10 perturbed samples of the plan-paired test set.

## Discussion

Among the configurations tested, the difference-target chain with full MPC integration showed the largest cumulative gain in GPR prediction. Within this chain, the addition of MPC features alone reduced the absolute GPR prediction error by approximately 20% relative to Base-D, while the further addition of MPCmod augmentation reduced it by an additional 28%, yielding a combined 42% reduction across the full chain. The two components thus appear to contribute in an approximately additive manner rather than redundantly, suggesting that they may act through distinct mechanisms: MPC features provide explicit machine-state information to the model, while deviation-aware augmentation exposes the model to a wider range of deviation scenarios during training. On unperturbed plans, MPC+aug-D and Base-D performed comparably on the fair-comparison test set, with no statistically significant difference in RMSE or Pearson’s r (**Table 4**), indicating that the augmentation strategy improved perturbation sensitivity without reducing accuracy on unperturbed plans. However, this combined gain was specific to the difference-target chain.

Within the EPID-target chain, the same stepwise additions yielded only a 21% combined reduction in absolute GPR prediction error, and adding MPC features alone reversed the polarity of the prediction error, with Base-E over-predicting the perturbed-plan GPR and MPC-E under-predicting it. This difference likely reflects that the dose-difference target isolates the dosimetric perturbations introduced by MLC offsets from the dominant background of the calculated dose. As a result, the model can learn the MPC–deviation mapping more directly than when predicting the full EPID intensity. The choice of prediction target may thus be a meaningful design factor, and its interaction with MPC conditioning and augmentation appears to shape the operating point of the approach.

Among prior deep learning approaches to PSQA prediction, the approach of Matsuura et al. (14) is the most directly comparable to the present work, as both employ Pix2Pix-based generative models in the EPID-based PSQA setting. The two approaches differ in their prediction target. Matsuura et al. synthesize the gamma distribution directly per criterion, which requires training a separate model for each criterion of interest, whereas the present approach yields the predicted EPID image, from which gamma analysis can be performed at any criterion at inference time. At the 3%/2 mm criterion, Matsuura et al. reported a pixel-level sensitivity of 51.9%, whereas the present approach achieved 29.6% on the fair-comparison test set. At the GPR level, the present approach achieved an RMSE of 0.48 pp and a Pearson’s r of 0.944, compared with 1.0 pp and 0.65 reported by Matsuura et al. under the same criterion. The two approaches thus appear complementary. Gamma-distribution synthesis may be favorable for pixel-level inspection under a fixed criterion, while EPID prediction may be favorable for accurate GPR estimation across multiple criteria. An additional feature of the present approach is the conditioning on daily MPC features, which allows the prediction to reflect the current state of the linac rather than relying solely on the planned dose distribution. The Pix2Pix backbone was adopted from this precedent, and keeping it fixed across all six variants ensures that the observed differences reflect the prediction target and the MPC conditioning rather than architectural factors. Whether more recent architectures could further improve performance within the same conditioning framework was not evaluated in this study and remains for future work.

A further point concerns the pixel-level sensitivity. Across the three criteria, the sensitivity did not exceed 32.18% (2%/2 mm) for any model variant, so about two thirds of the failing pixels were not identified. Predicting the GPR accurately and localizing the individual failing pixels are different tasks, and the present sensitivity is not sufficient for standalone localization of failure regions, which remains a role of physical measurement. The low sensitivity may arise because failing pixels form only a small fraction of the evaluation mask, and because a pixel counts as detected only when the predicted and measured failure regions overlap exactly, so even a small spatial shift is counted as a miss. Neither factor was investigated in this study. The multi-task formulation outlined in the future directions is aimed at narrowing this gap. The limited pixel-level overlap does not by itself exclude a plan-level screening role, however. Because GPR is routinely used as an operational summary metric in PSQA, a sufficiently validated prediction of the expected GPR could still identify plans whose estimated delivery quality has changed relative to their pretreatment baseline, even if the exact locations of all failing pixels are not identified. Such a signal would be adjunctive rather than diagnostic, prompting plan review, machine assessment, or repeat physical verification rather than a final pass/fail decision.

In its current form, the proposed approach is not intended to replace measurement-based PSQA, and physical measurement remains required for all plans. Rather, the approach could serve as a screening component within the existing verification workflow. Because it provides a fast, software-based estimate of both the predicted EPID image and the corresponding GPR prior to physical measurement, plans with a lower predicted GPR could be prioritized for early full verification. If non-inferior detection of clinically relevant delivery errors and an acceptably low false-negative risk were established, rapid automated calculation without phantom setup or treatment-machine occupancy would provide a clear operational advantage. The potential clinical role of the approach is, moreover, not limited to accelerating the single pretreatment assessment. Conventional measurement-based PSQA is generally performed once before the treatment course and therefore verifies the plan under the machine condition present at that time. Because MPC features are acquired daily, the expected GPR of an approved plan can in principle be re-evaluated against the updated machine state on the day each fraction is delivered, and an unexpected change relative to the pretreatment baseline could prompt additional plan review, machine assessment, or repeat physical verification. The same approach could support prioritized reassessment of ongoing treatment plans after relevant maintenance, calibration, or other machine-state changes. This concept would extend PSQA from a single pretreatment assessment toward fraction-level monitoring of patient-specific delivery quality during the treatment course. Such a prediction represents a machine-state-based prospective risk assessment. It does not verify the actual delivery of an individual fraction and does not account for intrafraction faults, patient setup, motion, or anatomical change; verification of fraction-specific delivery would require additional information such as trajectory-log, transit EPID (24), or *in vivo* dosimetry data. Software-based screening is further motivated by the two practical burdens of measurement-based PSQA noted in the Introduction. Repeated EPID exposure progressively accumulates dose on the panel, which can reach on the order of 10–20 kGy per year under frequent clinical use (25). Although the a-Si photodiodes themselves tolerate cumulative doses well above this range, the sensor leakage current begins to rise above a cumulative threshold of approximately 6 kGy (26). For the most heavily used linac at our institution, the imager accumulates approximately 11.5 kGy per year under the current PSQA workload, well above the 6 kGy threshold for sensor leakage onset. In addition, each PSQA session takes approximately 10 minutes and roughly 1,500 sessions are performed per linac per year, amounting to over 250 hours of measurement time annually. These burdens motivate the future prospective development of validated screening tools. The present retrospective proof-of-concept results do not by themselves establish clinical screening, plan prioritization, fraction-level use, or any reduction in measurement workload or detector exposure. Prospective validation with plan-level sensitivity, specificity, decision thresholds, and false-negative analysis, together with the temporal stability across the treatment course noted in the limitations, would be required before clinical application.

Several limitations should be acknowledged. First, the present evaluation is restricted to a single institution and a single Halcyon v3.0 system, and generalization to other linac platforms, including C-arm geometries, has not been tested. MPC is implemented within a vendor-defined QA framework, so the general concept of using measured machine-state information is not institution-specific; however, the available MPC parameters and their relationship to the EPID response may differ across machine platforms. The machine-state-to-EPID relationship learned here is tied to this Halcyon system, including its dual-layer MLC and imager, and the trained model should therefore not be transferred directly to another machine without machine-specific validation and, most likely, retraining. Whether the conditioning strategy generalizes across machines and institutions remains to be established. Second, although the patient cohort spans head-and-neck, pelvis, thorax, and extremities, pelvic cases (largely prostate) dominate the dataset, and broader site representation, particularly including stereotactic body radiation therapy (SBRT) of the lung and other small-volume targets, will be needed to characterize the approach’s behavior across the full clinical spectrum. Third, the perturbation-sensitivity evaluation rests on a small plan-paired test set of 16 samples, which limits statistical power. The observed differences should therefore be interpreted as exploratory rather than confirmatory. Fourth, the present evaluation establishes agreement between predicted and measured results rather than evidence for clinical implementation. A predicted GPR is not a physical delivery check, and prospective clinical validation has so far been reported only for a point-dose-based model (27), not for EPID-based image prediction. Therefore, the hybrid workflow described above should be regarded as illustrative. Its clinical use would require formal evaluation of decision thresholds, including plan-level pass/fail sensitivity, specificity, and false-negative risk, which was not performed in this study. Fifth, the synthetic-deviation dataset covers only MLC positioning offsets of a single magnitude. Real machine-state variations also include beam output drift, collimator rotation, imager calibration changes, and dynamic effects such as leaf speed errors, which were not represented. The masking analysis accordingly shows that the learned fault response resides in the MLC-related features, and whether the non-MLC MPC features would become informative under other fault types cannot be established from the present data. Expanding the deviation protocol toward these fault types is identified in the future directions.

Several future directions follow naturally from the present work. First, validation across multiple linac platforms (including other Halcyon and TrueBeam systems), institutions, and treatment sites (including SBRT of the lung and other small-volume targets), together with temporal validation across independent treatment periods and maintenance or calibration events, will be needed before the approach can be considered for clinical deployment. In addition, expanding the synthetic-deviation dataset to cover non-MLC machine perturbations would further characterize its operating envelope. Second, a multi-task formulation that simultaneously predicts both the EPID image and a tolerance-conditioned gamma map could potentially combine the GPR-prediction strengths of the present approach with the pixel-level failure localization strengths of gamma-synthesis approaches. Third, considering that MPC measurements are routinely acquired daily, integrating this workflow with an automated pipeline that updates its conditioning input at the start of each treatment day could represent a step toward machine-state-aware PSQA. Finally, because the present work establishes this concept retrospectively, a necessary next step is prospective evaluation of the approach alongside the current measurement-based workflow, in which the predicted results are assessed without altering clinical decisions. Such an evaluation should assess plan-level sensitivity, specificity, false-negative risk, and temporal stability, and whether a change in the machine-state-conditioned predicted GPR can reliably identify clinically relevant machine-state changes. Following such validation, the method could be refined and made available as a reproducible framework.

## Conclusion

This study tested the hypothesis that incorporating daily machine-state information into EPID-based PSQA prediction can improve the estimation of patient-specific delivery quality. The results demonstrate that MPC features, used as conditioning inputs together with deviation-aware augmentation, improved the prediction of EPID images and the corresponding GPRs under perturbed delivery conditions. These findings support a new concept of machine-state-aware PSQA, in which evaluation is not limited to a single pretreatment measurement of the approved plan but could be extended toward assessing delivery quality for individual fractions during fractionated radiotherapy. Rather than replacing measurement-based PSQA, this approach offers a complementary strategy that may broaden verification from the plan level to a fraction-level, machine-state-aware PSQA.

## Data Availability

The original contributions presented in the study are included in the article/supplementary material. Further inquiries can be directed to the corresponding authors.

## References

[1] International Commission on Radiation Units and Measurements (ICRU) . Prescribing, recording, and reporting photon-beam intensity-modulated radiation therapy (IMRT) (ICRU Report 83). J ICRU. (2010) 10:1–106. doi: 10.1093/jicru/ndq002

[2] Miften M Olch A Mihailidis D Moran J Pawlicki T Molineu A . Tolerance limits and methodologies for IMRT measurement-based verification QA: Recommendations of AAPM Task Group No. 218. Med Phys. (2018) 45:e53–83. doi: 10.1002/mp.12810 29443390

[3] Dogan N Mijnheer BJ Padgett K Nalichowski A Wu C Nyflot MJ . AAPM task group report 307: use of EPIDs for patient-specific IMRT and VMAT QA. Med Phys. (2023) 50:e865–903. doi: 10.1002/mp.16536 37384416 PMC11230298

[4] Kry SF Glenn MC Peterson CB Branco D Mehrens H Steinmann A . Independent recalculation outperforms traditional measurement-based IMRT QA methods in detecting unacceptable plans. Med Phys. (2019) 46:3700–8. doi: 10.1002/mp.13638 31152568 PMC6692191

[5] Agnew CE Irvine DM McGarry CK . Correlation of phantom-based and log file patient-specific QA with complexity scores for VMAT. J Appl Clin Med Phys. (2014) 15:204–16. doi: 10.1120/jacmp.v15i6.4994 25493524 PMC5711124

[6] Valdes G Scheuermann R Hung CY Olszanski A Bellerive M Solberg TD . A mathematical framework for virtual IMRT QA using machine learning. Med Phys. (2016) 43:4323–34. doi: 10.1118/1.4953835 27370147

[7] Valdes G Chan MF Lim SB Scheuermann R Deasy JO Solberg TD . IMRT QA using machine learning: A multi-institutional validation. J Appl Clin Med Phys. (2017) 18:279–84. doi: 10.1002/acm2.12161 28815994 PMC5874948

[8] Lam D Zhang X Li H Deshan Y Schott B Zhao T . Predicting gamma passing rates for portal dosimetry-based IMRT QA using machine learning. Med Phys. (2019) 46:4666–75. doi: 10.1002/mp.13752 31386761

[9] Zhu H Zhu Q Wang Z Yang B Zhang W Qiu J . Patient-specific quality assurance prediction models based on machine learning for novel dual-layered MLC linac. Med Phys. (2023) 50:1205–14. doi: 10.1002/mp.16091 36342293

[10] Huang Y Pi Y Ma K Miao X Fu S Chen H . Virtual patient-specific quality assurance of IMRT using UNet++: Classification, gamma passing rates prediction, and dose difference prediction. Front Oncol. (2021) 11:700343. doi: 10.3389/fonc.2021.700343 34354949 PMC8330420

[11] Yoganathan SA Ahmed S Paloor S Torfeh T Aouadi S Al-Hammadi N . Virtual pretreatment patient-specific quality assurance of volumetric modulated arc therapy using deep learning. Med Phys. (2023) 50:7891–903. doi: 10.1002/mp.16567 37379068

[12] Dal Bosco L Franceries X Romain B Smekens F Husson F Le Lann MV . A convolutional neural network model for EPID-based non-transit dosimetry. J Appl Clin Med Phys. (2023) 24:e13923. doi: 10.1002/acm2.13923 36864758 PMC10243321

[13] Tozuka R Kadoya N Tomori S Kimura Y Kajikawa T Sugai Y . Improvement of deep learning prediction model in patient-specific QA for VMAT with MLC leaf position map and patient's dose distribution. J Appl Clin Med Phys. (2023) 24:e14055. doi: 10.1002/acm2.14055 37261720 PMC10562023

[14] Matsuura T Kawahara D Saito A Yamada K Ozawa S Nagata Y . A synthesized gamma distribution-based patient-specific VMAT QA using a generative adversarial network. Med Phys. (2023) 50:2488–98. doi: 10.1002/mp.16210 36609669

[15] Isola P Zhu JY Zhou T Efros AA . Image-to-image translation with conditional adversarial networks. In: Arxiv Preprint. Ithaca, NY, United States: arXiv, Cornell University (2017). doi: 10.48550/arXiv.1611.07004

[16] Goodfellow IJ Pouget-Abadie J Mirza M Xu B Warde-Farley D Ozair S . Generative adversarial networks. In: Arxiv Preprint. Ithaca, NY, United States: arXiv, Cornell University (2014). doi: 10.48550/arXiv.1406.2661

[17] Mirza M Osindero S . Conditional generative adversarial nets. In: Arxiv Preprint. Ithaca, NY, United States: arXiv, Cornell University (2014). doi: 10.48550/arXiv.1411.1784

[18] Paszke A Gross S Massa F Lerer A Bradbury J Chanan G . PyTorch: An imperative style, high-performance deep learning library. In: Arxiv Preprint. Ithaca, NY, United States: arXiv, Cornell University (2019). doi: 10.48550/arXiv.1912.01703

[19] Li Y Netherton T Nitsch PL Gao S Klopp AH Balter PA . Independent validation of machine performance check for the Halcyon and TrueBeam linacs for daily quality assurance. J Appl Clin Med Phys. (2018) 19:375–82. doi: 10.1002/acm2.12391 30016578 PMC6123154

[20] Klein EE Hanley J Bayouth J Yin FF Simon W Dresser S . Task Group 142 report: quality assurance of medical accelerators. Med Phys. (2009) 36:4197–212. doi: 10.1118/1.3190392 19810494

[21] Low DA Harms WB Mutic S Purdy JA . A technique for the quantitative evaluation of dose distributions. Med Phys. (1998) 25:656–61. doi: 10.1118/1.598248 9608475

[22] Biggs S Jennings M Swerdloff S Chlap P Lane D Rembish J . PyMedPhys: a community effort to develop an open, Python-based standard library for medical physics applications. J Open Source Softw. (2022) 7:4555. doi: 10.21105/joss.04555

[23] Virtanen P Gommers R Oliphant TE Haberland M Reddy T Cournapeau D . SciPy 1.0: fundamental algorithms for scientific computing in Python. Nat Methods. (2020) 17:261–72. doi: 10.1038/s41592-019-0686-2 32015543 PMC7056644

[24] Baek TS Chung EJ Son J Yoon M . Feasibility study of patient specific quality assurance using transit dosimetry based on measurement with an electronic portal imaging device. Prog Med Phys. (2017) 28:54–60. doi: 10.14316/pmp.2017.28.2.54

[25] Antonuk LE . Electronic portal imaging devices: a review and historical perspective of contemporary technologies and research. Phys Med Biol. (2002) 47:R31–65. doi: 10.1088/0031-9155/47/6/201 11936185

[26] Boudry JM Antonuk LE . Radiation damage of amorphous silicon photodiode sensors. IEEE Trans Nucl Sci. (1994) 41:703–7. doi: 10.1109/23.322792

[27] Wall PDH Hirata E Morin O Valdes G Witztum A . Prospective clinical validation of virtual patient-specific quality assurance of volumetric modulated arc therapy radiation therapy plans. Int J Radiat Oncol Biol Phys. (2022) 113:1091–102. doi: 10.1016/j.ijrobp.2022.04.040 35533908

